# Unveiling nuances in data analysis to illuminate marine pilot strain

**DOI:** 10.3389/fpsyg.2024.1417215

**Published:** 2024-09-03

**Authors:** Andrej Košir, Matija Svetina, Marko Perkovič, Franc Dimc, Tanja Brcko, Dejan Žagar

**Affiliations:** ^1^Faculty of Electrical Engineering, University of Ljubljana, Ljubljana, Slovenia; ^2^Faculty of Arts, Department of Psychology, University of Ljubljana, Ljubljana, Slovenia; ^3^Faculty of Maritime Studies and Transport, University of Ljubljana, Portorož, Slovenia

**Keywords:** pilotage, port approach, simulation, risk assessment, cognitive load, physiological response, data averaging

## Abstract

Maritime studies, encompassing a range of disciplines, increasingly rely on advanced data analytics, particularly in the context of navigation. As technology advances, the statistical averaging of large datasets has become a critical component of these analyses. However, recent studies have highlighted discrepancies between statistical predictions and observable realities, especially in high-stress environments like port approach procedures conducted by marine pilots. This study analyzed physiological responses recorded during simulation exercises involving experienced marine pilots. The focus was not on the specific outcomes of the simulations but on the potential faults arising from conventional statistical signal processing, particularly mean-centered approaches. A large dataset of signals was generated, including one signal with a dominant characteristic intentionally designed to introduce imbalance, mimicking the uneven distribution of real-world data. Initial analysis suggested that the average physiological response of the pilots followed an S-shaped curve, indicative of a psycho-physiological reaction to stress. However, further post hoc analysis revealed that this pattern was primarily influenced by a single participant’s data. This finding raises concerns about the generalizability of the S-curve as a typical stress response in maritime pilots. The results underscore the limitations of relying solely on conventional statistical methods, such as mean-centered approaches, in interpreting complex datasets. The study calls into question the validity of standardizing data interpretations based on dominant characteristic curves, particularly in environments as intricate as maritime navigation. The research highlights the need for a re-evaluation of these methods to ensure more reliable and nuanced conclusions in maritime studies. This study contributes to the ongoing discourse on data interpretation in maritime research, emphasizing the critical need to re-assess conventional statistical signal processing techniques. By recognizing the potential pitfalls in data generalization, the study advocates for more robust analytical approaches to better capture the complexities of real-world maritime challenges.

## 1 Introduction

This paper discusses the reasons for misleading conclusions in scientific texts, generated during postprocessing through unintentional presumption or by basic error. These types of results provide deceptive information that can cause misleading representations of subjects and doubts about methodology. The article aims to highlight the challenges of the statistical approach in analysing physiological data that we faced in the risk assessment of ship simulation during port approach and berthing (Žagar et al., 2022). Uncritical data summarization typically happens due to oversimplifying complex information, ignoring essential details, or presenting a biased perspective, witnessed in politics and advertising, where at least one of the following factors is included (Calzon, 2023):

- Sampling bias when the sample used in the study does not represent the population being studied.
- Confounding variables when the influencing factors are not accounted for in the analysis.
- Incorrect statistical methods when the approach must accurately reflect the relationship between the variables.
- Hypothesis errors when the null hypothesis is rejected even though it is accurate or vice versa.
- Small sample size when more statistical information is required to detect significant effects.
- Publication bias is when an overestimation of the proper effect size leads to significant results which are more likely to be published.

To avoid misleading conclusions, it is essential to consider the context and nuances of the information and any potential sources of error or bias to avoid flawed conclusions and misguided suggestions. The impetus of this paper comes largely from the need to fill a significant gap regarding the working conditions of pilots.

The profession of sea pilot is demanding, pilots consistently exposed to irregular work and sleep schedules, extreme and concentrated temperature extremes, adverse weather conditions, and frequent exposure to unfamiliar, stressful, and high-risk work environments. Individuals who work continuously in such settings for long periods often develop risky behaviors. Once risky behaviors are established in seafarers, decision-making becomes impaired, and the risk of maritime accidents increases (Xu et al., 2021). Decision-making is consistently cited as one of the essential factors in shipping accidents or incidents and is critical in pilotage operations (Butler et al., 2022). On top of that, solutions are generally abstract and technological, not natural. For instance, avoiding a head-on collision was once a simple matter of both parties turning right, something natural and simple; now navigation itself is quite abstracted, involving instruments that already remove the pilot from the natural plane of human reaction.

In marine cognitive load and stress analysis, researchers commonly analyse data related to performance measures, physiological data, or subjective ratings of workload with the mean performance score of participants under different workload conditions calculated to compare cognitive load between various tasks (Table 1). Researchers face the foundational challenge of determining central value representatives while analysing their data. The selection of an appropriate statistical approach is contingent upon the nuances of the research question and the characteristics of the analysed dataset.

**Table 1:** Review of cognitive load and stress analysis.

Stress risk	Year	Sensor type	Author	Participants
1	1998	Review	AMSA (1998)	Pilots
2	1999	Review	Lovell (1999)	Pilots
3	2015	Review	Main et al. (2017)	Pilots
4	1990	Psychomotor task	Shull (1990)	Pilots
5	2020	Questionnaire	Maglić et al. (2020)	OOWs
6	2018	EEG-stress	Lim et al. (2018)	Pilots
7	2017	EEG-emotions	Liu et al. (2024)	Students
8	2016	Self-assessment, TLX	Di Nocera et al. (2016)	OOWs
9	2019	Cardiovascular, TLX	Barbarewicz et al. (2019)	Pilots
10	2019	EDA	Zontone et al. (2019)	Students
11	2019	Pupil, bio response	Barbarewicz et al. (2019)	Pilots
12	2021	Questionnaire	Oldenburg et al. (2021)	Pilots
13	2022	Questionnaire	Butler et al. (2022)	Pilots
14	2021	Questionnaire	Xu et al. (2021)	Pilots
15	2022	Lidar	Kang et al. (2022)	Pilots
16	2014	Questionnaire	Ceyhun and Ozbag (2014)	Pilots
17	2022	EDA, HR	Žagar et al. (2022)	Pilots, students
Cognitive load				Tug masters
18	2007	ECG, TLX	Kim et al. (2007)	Students vs. pilots
19	2010	Eye-tracking	Arenius et al. (2010)	Students
20	2010	Self-report	Jha et al., 2010)	Us marines
21	2012	ECG electrodes	Saus et al. (2012)	Students
22	2015	Self-report	Haase et al. (2015)	Elite athletes
23	2016	Eye-tracking	Hareide and Ostnes (2017)	Officers
24	2018	Self-assessment, TLX	Orlandi and Brooks (2018)	Pilots
25	2018	Pupil	Fridman et al. (2018)	Drivers
26	2020	HR, BVP	Kim (2010)	OOWs

The question, therefore, is what kind of sample averaging has typically been used when studying a bridge task performance. To determine approaches typically used to study cognitive load and stress risk we analysed the postprocessing approach in 23 published articles:

The common ground of the studies listed in Table 1 is the determination of the causes of cognitive load and stress in the maritime and transportation sectors by measuring physiological responses while participants performed a simulation. The results generally showed that cognitive load was highest when participants had to process a large amount of information and make quick decisions. Hence, task complexity significantly affected cognitive load.

The analysis showed that 34% of authors used standard methods of data analyses, averaging large amounts of physiological data and identifying trends or patterns during post-processing analyses; ANOVA is used in 22%; regression analyses are used in 13%; SPSS and support vector classifier in 9% each, neural networks 4%, and undefined methods in 9%.

The problem with identifying trends or patterns by averaging large amounts of physiological data is the tendency to disregard significant variability and differences between individual data points. Averaging can also mask essential outliers or subgroups within the data, which may have unique characteristics and require separate analysis: Simpson’s paradox (Geng, 2011). The effect is driven by a subgroup with a specific characteristic (e.g., introverted, aggressive, experienced participants). Averaging the data across all individuals can mask this critical finding. The question is, what can be done to recognize and avoid this challenge in physiological data? The following section provides a brief overview of this topic.

## 2 Problem statement and goal

This article addresses data from two sources: physiological data from six pilots simulating complex port approach procedures, as reported in (Žagar et al., 2024), and the expanded amount of data into a larger sample. Challenges faced in analysing and interpreting real-world data and resolutions were clearer.

An empirical study (Žagar et al., 2024) compared the physiological responses of experienced marine pilots and trainees. The average response on a small sample of experienced pilots (*n* = 6) yielded an approximate S shape, indicating that a psychophysiological reaction precedes a stress event. A detailed post-festum examination of individual responses, however, revealed that only one of six participants matched this pattern, whereas the psychophysiological responses of the other participants were relatively flat. Detailed post-festum analysis showed that the characteristic of the S shape obtained by averaging was determined mainly by one participant rather than mirroring the typical response pattern in the group of participants. Although averaging the data is correct, the detailed post-festum analysis suggested that generalizing the S curve as a typical psychophysiological response to stress might be misleading.

The phenomenon causing misinterpretation occurs when there is a low number m of S curves compared to the number of curves n. Note however that when m is not small according to n, the resulting mean S shape curve would not be an outlier but the result of a regular experimental outcome. In this case, the mean S shape would be the correct conclusion rather than a result of misinterpretation.

**Figure 1:**
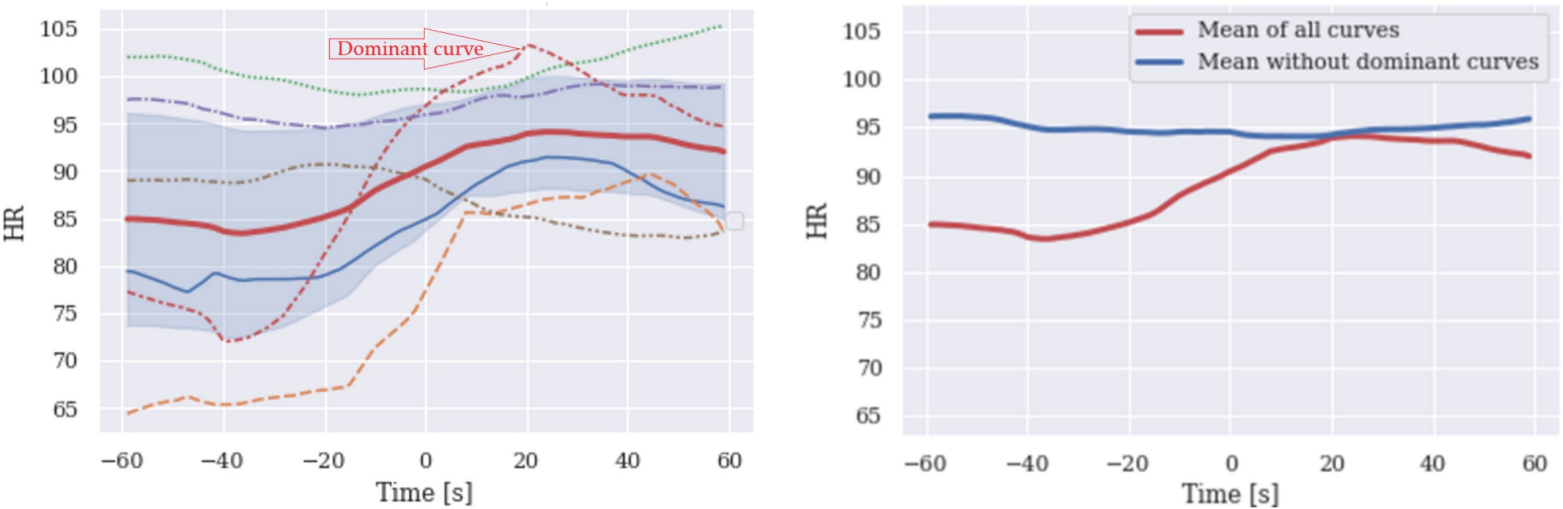
S shaped mean of six HR curves (left). The dominant characteristic curve determines the shape of the HR mean curve, and the rest contribute much less to the shape. Averaging non-characteristic curves yields no characteristic shape (right, blue), while the average of all six curves has a precise S shape. This may be misleading since only one curve seems to determine the mean curve.

The problem is that the estimation of confidence intervals would not always indicate that there is a hidden dominant response. Furthermore, the resultant estimated confidence interval (depicted as the blue area in Figure 1) may narrow when other non-characteristic curves exhibit a nearly flat signal. This phenomenon could lead to biased conclusions like those drawn from the predominant S shape, highlighting the importance of scrutinizing the broader context and considering potential confounding factors in data interpretation. The distortion may primarily be related to relatively high inter-individual variability and a small sample size. To test this assumption, we simulated the data on larger sample sizes, addressing the difficulty of possible misleading inferences from psychophysiological signals in the context of a sample size to provide guidelines for identifying subgroups of data that may determine the response pattern and propose a solution to this problem.

## 3 Materials and methods

In processing our data, we were confronted with the misleading aspect that averaging may lead to potentially erroneous conclusions as a typical statistical approach, post-processing showing that:

- The relationship between variables is non-linear and averaging can lead to incorrect conclusions. This might happen in scenarios where the relationship between the participant’s experience and the demanding cognitive task varies, and the averaging of the data might suggest a linear relationship that does not accurately represent the data.
- Outliers can significantly impact the averaging results, leading to potentially incorrect conclusions, where a single data point is very different from the rest of the data, leading to inaccurate conclusions.
- In scenarios where participants vary in characteristics, averaging the data may obscure important differences between groups. The significant variation within the data set (heterogeneity) is not accurately captured.
- If the data is collected through a biased sampling approach, averaging can lead to incorrect conclusions, especially in the case when participants from a specific non-representative group (students and cadets) reflect (falsely) the larger population (experienced mariners).

Thus, to avoid potentially incorrect conclusions due to averaging, careful consideration of the data and statistical methods during post-processing is necessary, using alternative approaches, such as visualization and non-parametric tests, applicable to the specific data and research question. Following, we explain the design of both the experimental and the generated data.

### 3.1 Real-world data

The experimental data are from the test conducted in Wartsila’s Navi Trainer 5,000 ship handling simulator. The full-mission simulator consists of a modern and ergonomic navigation console with standard navigation equipment, such as redundant multifunctional displays, a conning station, a ship’s wheel stand, an overhead monitor and a communication unit. The simulator offers hundreds of vessels for creating the most complex scenarios in different weather conditions and navigation areas. The simulator is also equipped with visualization and provides a 270° view of the scene. All activities performed by the pilots were recorded.

The Empatica E4 sensor obtained physiological data during the experiment. Physiological data were obtained from six participants during a simulated port approaching procedure wherein a large container ship was heading to a designated berth. We collected and processed various signals, including the participants’ average heart rate (HR), sampled at 1 Hz. Each average HR value was computed from inter-beat intervals (IBI) within 10-s spans. Electrodermal activity (EDA) was sampled at 4 Hz for this study. Further details on experimental scenarios and procedures are available in our previous publication (Žagar et al., 2024). Due to constraints associated with the availability of experienced marine pilots, our real-world dataset needed to be improved. We conducted simulations to address this limitation and enrich the dataset with data from the earlier work.

### 3.2 Simulated data

In addition to the data obtained in the simulator, computer simulations obtained random data based on a real data model, using continuous autoregressive signal generation where the data model was estimated from real data (see section 3.1). For this purpose, the Python library TimeSynth^1^ ver. 0.2.4 was run to obtain 110 simulated time series of HR sampled at 1 Hz and EDA sampled at 4 Hz separately sampled at the same frequency as real signals. The number of signals is driven by the need for a diverse dataset that includes a variety of simulated time series’. In statistical analysis and machine learning, having a sufficiently large and diverse dataset is crucial for obtaining robust and results fit for generalization. In this context, using 110 simulated heart rate (HR) time series’ and electrodermal activity (EDA) enables a comprehensive exploration of different signal characteristics. All but one of these signals were generated from the “no shape group” and one from the “shape group.” The “no shape” group’s signals have no characteristic shape and vary little. The “shape” group consists of signals that have a characteristic shape with higher total variation, see section 3.2.1.

Assuming a set of discrete time-dependent psychophysiological signals is denoted by S , where some of them belong to a “no shape group” $S_{a}$ (they average to a flat curve) and some of them to a “characteristic shape” group $S_{b}$ (they average into a characteristic shape). In our notation, $S=S_{a}\cup S_{b}$ . The average signal of a given set of signals S is denoted by $\mu_{S}$ = $\frac{1}{\left|S\right|}\underset{s\in S}{\sum }s\text{,}$ where $\left|S\right|$ is the number of elements of the set. The assumption is that the signal $\mu_{S_{a}}$ has no shape (i.e., close to a flat curve) and the signal $\mu_{S_{b}}$ has a characteristic shape used to illustrate the phenomenon, leading to the issue we address in this paper manifest in the following two situations:

- A set of signals comprises one or a few characteristic shape curves and numerous close-to-flat curves.
- A set of signals comprises one or a few characteristic shape curves and numerous random curves.

The conclusion regarding the pattern (in our case, the expectation of stressful events by the marine pilot can be seen from the psychophysiological signals) is typically made on the average of signals (curves). Formally, we can break down the average curves as a weighted sum of averaged signals to


$$\mu_{S}=\frac{\left|S_{a}\right|}{\left|S\right|}\;\mu_{S_{a}}+\frac{\left|S_{b}\right|}{\left|S\right|}\;\mu_{S_{b}}\text{.}$$

Even though the weight $\frac{\left|S_{b}\right|}{\left|S\right|}$ of the signal group $S_{b}$ is much smaller than the weight of the “no shape” group $S_{a}$ , the shape of $\mu_{S_{b}}$ determines the shape of the all-signal average $\mu_{S}\text{.}$ Compare this reasoning to results given in Sec 4. Results.

#### 3.2.1 Total variation as a measure of an impact on the mean curve

The total variation of the curve can be used to measure how dominant one curve is in terms of its impact on the mean curve. A time-dependent signal s on a time interval $\left[a,b\right]$ is given by


$$V_{a}^{b}\left(s\right)=\underset{P}{\sup}\overset{n_{P}-1}{\underset{i=0}{\sum }}\left|s\left(t_{i+1}\right)-s\left(t_{i}\right)\right|$$

for all partitions $P=\left\{t_{0},t_{1},\ldots ,t_{n_{P}}\right\}$ and interval $\left[a,b\right]$ where sup is a supremum over all partitions. If the partition P is selected such that the signal s is monotone on any time interval $\left[t_{i},t_{i+1}\right]$ , the total variation is $V_{a}^{b}\left(s\right)$ simply a sum of absolute differences without the supremum. To allow comparisons, we normalize total variation to a 1-s interval; this normalized total variation is TV divided by the time interval lengths in seconds.

The total variation adds all the highs and lows of the curve. Near-flat curves have TV values close to 0. For HR signals, HR 1 to HR 6 as shown in Figure 1 (left) normalized TVs are 0.196, 0.285, 0.102, 0.383, 0.074, 0.093. Observe that the lowest TV has the signal HR 5, closest to the flat curve, and the highest normalized TV has the signal HR 4, which has the most notable shape.

#### 3.2.2 Detecting the hidden anomaly

How can the researchers know when this anomaly might affect the findings? Practically speaking, the challenge is that modern signal plotting libraries (such as Python Matplotlib) do the normalization of axis on their own and thus hide the underlying challenge. There are a couple of methods, however, that may help the researchers detect the hidden determinator:

- Visualization: a group visualization of all signals and their average in the same plot and carefully examining their shapes. If the researcher is aware of the potentially misleading conclusion, it will be clear.
- Confidence interval (CI) estimation: if the confidence interval of the average is estimated and visualized, the analysis can indicate that a hidden determinator might occur. CI interval estimation can help the researcher when the reason for the average flattening is that the signals are close to flat and not when the reason is that the signals are random.
- Bootstrap and total variation procedures: A histogram of total variations generated by the bootstrap method can help identify a hidden determinator. When there is no dominant small subset of curves, the histogram is unimodal, and when there is a dominant subgroup, the histogram will be bimodal. This relates to the fact that bootstrap samples that contain no dominant curves will compose a major part of the histogram, but those with dominant ones will add a separate component of higher values.

#### 3.2.3 Modeling the S-curve formation process

The aim of modeling S-curve formation is to show that the occurrence of a small number of S-curves might be a regular rather than irregular event. In this case, an S-curve should not be treated as an outlier.

To provide further insight into the problem of misinterpretation of S-curve occurrences, we modeled the process of S-curve creation to show that S curves are not outliers but may appear in a regular experimental (and work-task) process.

The rise of the measured physiological signal (the response curve has an S shape) occurs when the number of stress circumstances faced by the participant is large enough to initiate a stress response (this assumption is further discussed in section 5). A normalized S curve of a single participant is modeled by the time it starts to rise, denoted by $t_{r}$ . The probability distribution of number events required to initiate the stress response is geometrical with parameter p where p is the probability that the event occurs in one attempt. The assumption behind stress event responses’ independence is also discussed in section 5. In our experiment, times of and nature of stressful events were the same for all participants. In particular, there were two such events built into the experiment, and therefore two possible response curves $\left\{s_{i}:i=1,2\right\}$ }. In the model, the probability of occurrence of the response curve (a measured physiological signal for a given pilot) was obtained by the geometrical distribution. Theoretically, S-curve as a response to the stressor, may occur after the threshold stress was achieved, and might be different in different individuals. Therefore, in some participants, S-curve as a response to the stressor may occur after the first stress event, or after several stress-related events have been present one after another. Our approach accounts for these inter-individual differences. In the current paper, we assumed that the probability of getting stressed in a single stressful circumstance (where the response shows S shape) can be modeled for a given participant or a group of participants.

The parameter of geometrical distribution can be estimated from the data representing either students (denoted by $p_{s})$ , or experienced pilots (denoted by $p_{p})$ . To demonstrate the estimation on our experimental data, we can estimate these probabilities by counting the number of S curve appearances at each of the stressful events. In particular, for two stressful events that occurred at times *t* $\left\{t_{i}:i=1,2\right\}$ , the counts are denoted by *k* $\left\{k_{i}:i=1,2\right\}$ }., respectively. An overdetermined system of nonlinear equations for a distribution parameter using these counts can be set and solved using square error minimization. There were 8 students and 8 pilots involved in the experiment with two stressful events and we obtained $p_{s}=0.14$ and $p_{p}=0.06\text{.}$ Note that this procedure of estimation may be applicable for an arbitrary sequence of stressful events.

## 4 Results

Here, misleading curves regarding two psychophysiological signals are illustrated: heart rate (HR) and electro-dermal activity (EDA). Since the number of signals available was very low, we generated simulated curves based on models from real data.

### 4.1 Measured signals

HR results are depicted in Figure 1. Note that three out of six curves impose the shape of the mean. Because of the low number of curves, the confidence interval cannot detect an anomaly; the visualization does.

**Figure 2:**
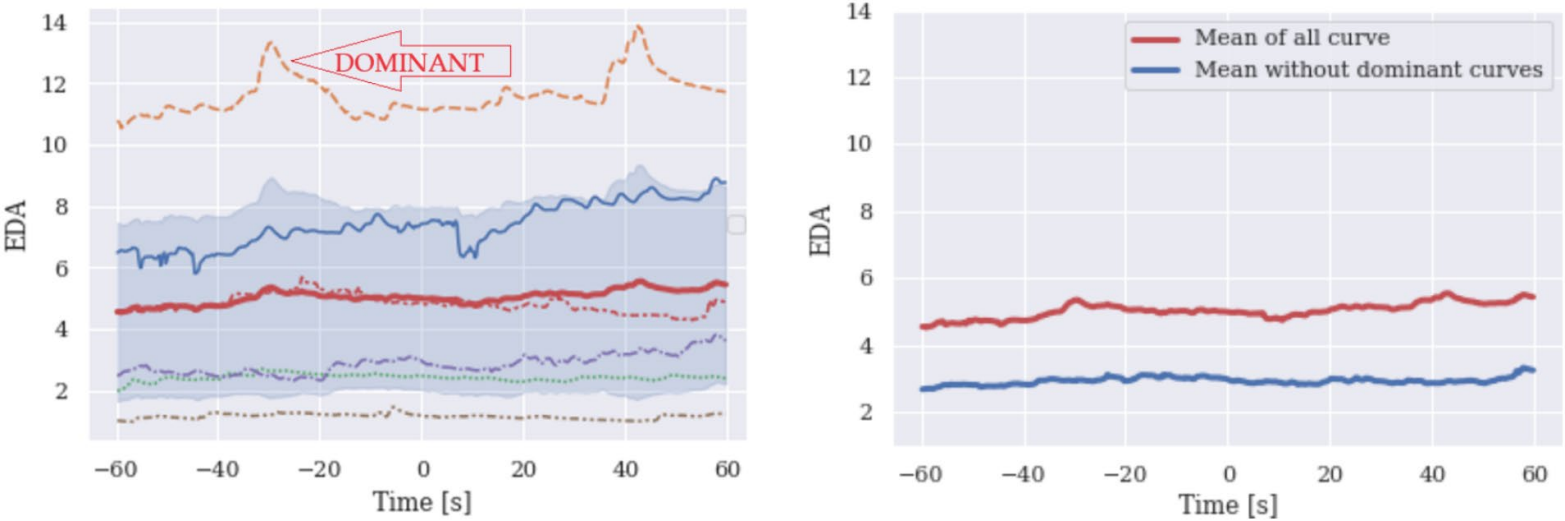
EDA: The orange curve on the left figure is dominant. The right Figure shows the mean of the EDA curve (plotted on the left side). The upper red curve presents the mean of all curves and blue presents the mean without a dominant curve.

EDA signal results are shown in Figure 2. According to the curve shapes, the effect of the characteristic curve imposing the shape is low, but we still observe that a single curve dominates the shape of the curve mean. For the same reason, this can be identified primarily by plotting the curves and visually inspect individual data.

The identification of an anomaly is based on the bootstrap procedure, which is not very effective with a low number of signals.

### 4.2 Simulated case signals

To demonstrate the effect of a small number of dominant curves imposing the shape of the mean of all curves and to explain how we identify the challenge, we randomly generated larger samples of HR and EDA signals.

#### 4.2.1 Identification using signal visualization

**Figure 3:**
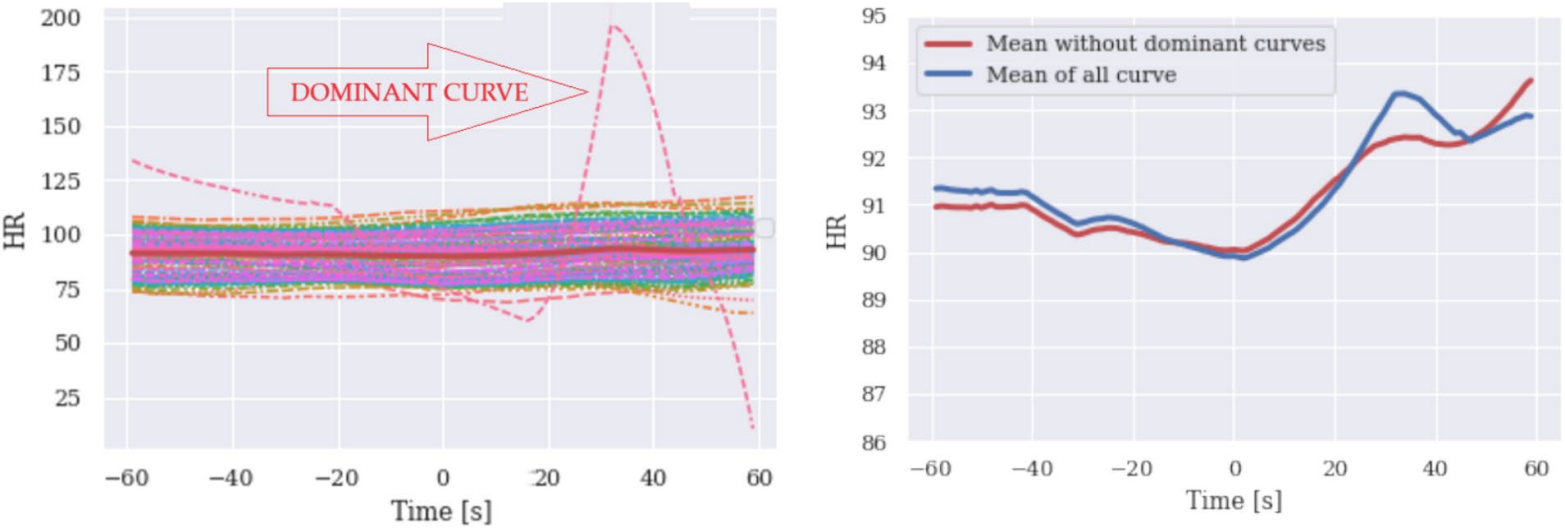
Simulated heart rate responses are given with their mean (left). Means of all signals are reproduced (right, red curve) for purposes of comparison.

**Figure 4:**
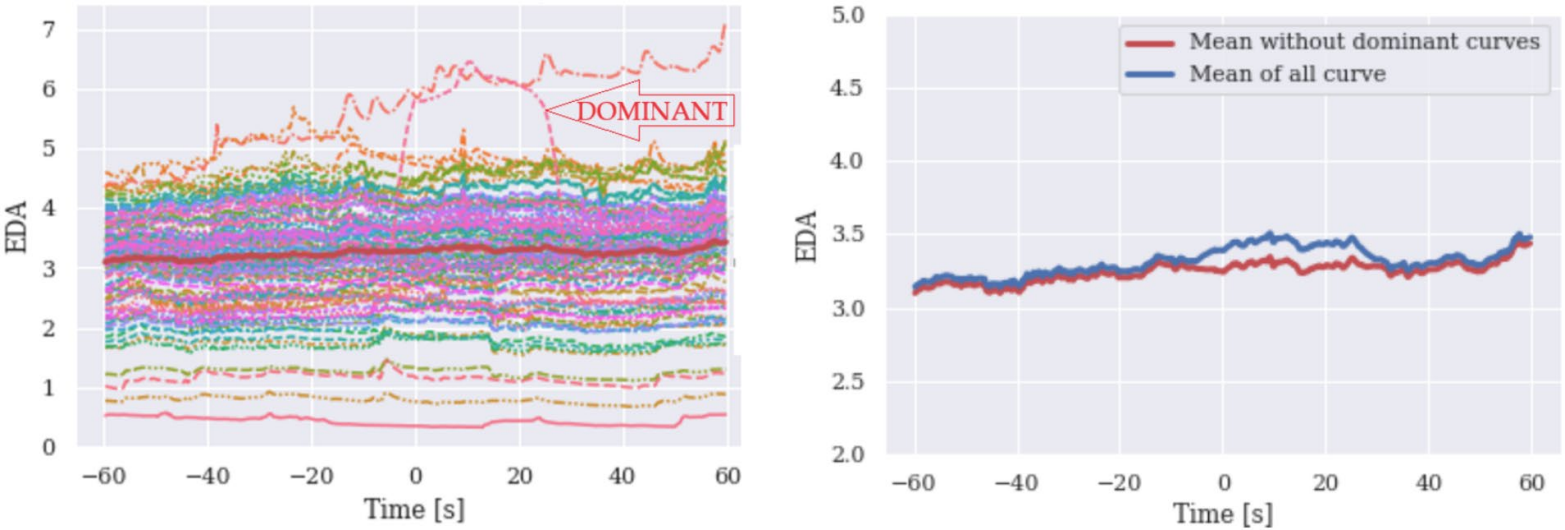
Simulated EDA are given with their mean (left). Means of all signals are reproduced for purposes of comparison.

On the left side of Figure 3, a series of HR signals and their mean value curve are plotted, with a dominant signal that deviates considerably. On the right side of the graph, the two mean curves are shown, one of which does not contain the dominant hard rate signal (red signal). The dominant signal strongly influences the mean value of all measurements. To identify these hidden determinator curves, the confidence interval (CI) can be used in addition to plotting and visual inspection. Comparing the curves, we observe that there are curves of different shapes compared to others and this should alert the researcher to the possibility that the mean curve may be inaccurate. Note that CI alone does not indicate any anomaly. Simulated data of HR and EDA are presented in Figures 3, 4, respectively.

#### 4.2.2 Identification using TV and bootstrap

**Figure 5:**
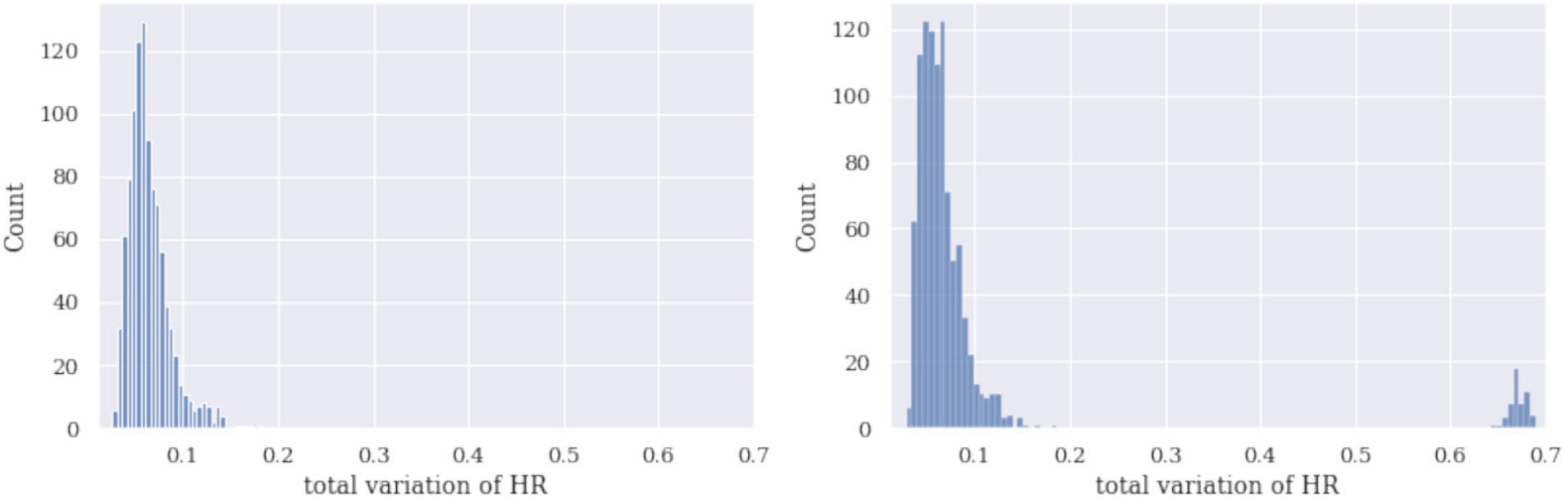
Histograms of mean TVs using a bootstrap procedure of simulated HR are given: no dominant curve (left) and with the dominant curve (right). Note that the dominant curve reflects two components on the histogram on the right.

**Figure 6:**
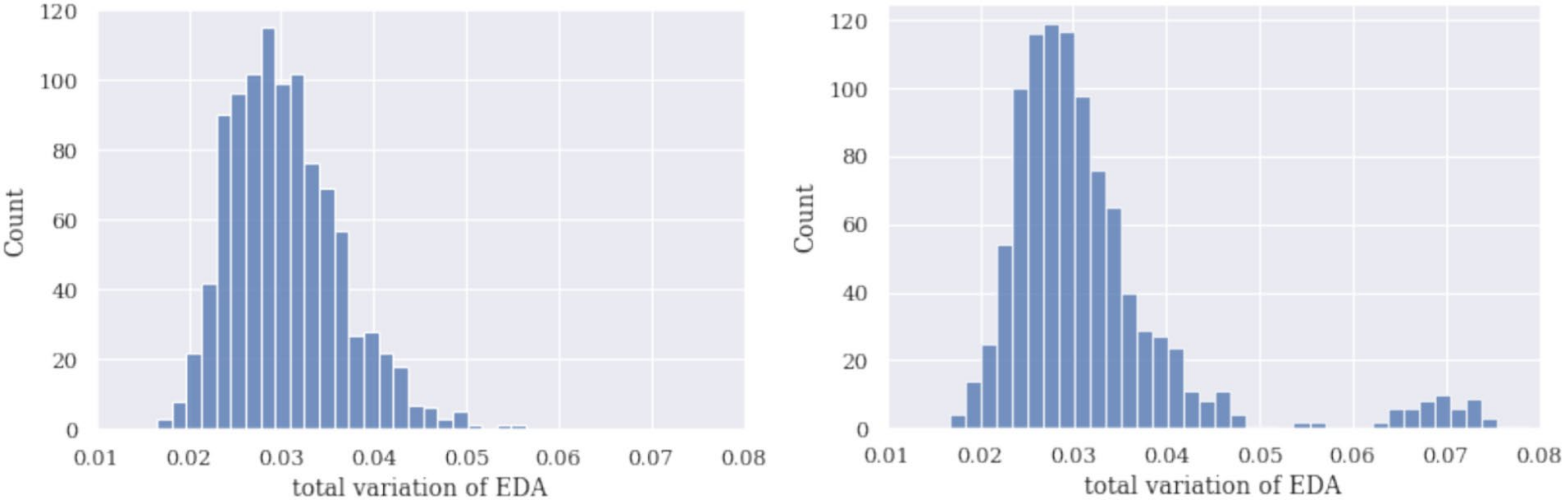
Histograms of sample mean TVs using a bootstrap procedure of simulated EDA are given without the dominant curve (left) and with the dominant curve (right). Again, the histogram has two components as a dominant curve is reflected.

The third approach to identifying hidden determinators is bootstrapping. To demonstrate the challenge of the identification approach using total variation (TV) and bootstrap, we generated histograms of TVs for simulated signals HR and EDA with and without a dominant subset of signals. In the bootstrap procedure, a set of 110 curves was sampled *n* = 1,000 times with sample sizes 5, first without and then with a dominant curve. The TV of a sample mean curve was calculated, and the histograms of these mean TVs are reported in Figures 5, 6. The sample size should be small enough for the dominant curves to impact the sample mean and make them distinguishable on the histogram.

Figures 5, 6 demonstrate that the bimodal (or polymodal) histograms can identify a dominant curve. If several types of shapes were present in the set of signals, they would appear on the sample TV histogram as several separate components. In the signals shown above, two types of shapes (no characteristic or dominant shape) and two components can be seen. Several bootstrap runs not reported here showed that the effect on the histogram (including one versus several components) is stable.

## 5 Discussion

The data used to analyse statistical readings are based on two sources. The first data source comes from experiments with marine pilots, where we collected physiological data (heart rate and electrodermal activity) from experienced pilots during a port approach in a full-mission simulator. Further considerations are based on the data from six pilots; the averaged results of their responses revealed an approximate S-shape, suggesting that a psychophysiological reaction likely precedes stressful events. In a detailed post-festum analysis that followed individual data sets were plotted to link them to the onset of the stress event. This visualization showed that the S-curve likely reflected the pattern of a single pilot. In contrast, the patterns of the other five participants, which were relatively flat, had a smaller effect on the averaged curve. This appears to be a false indication because the response of a single pilot determined the average for the entire group.

The first attempt to correct results was to treat this S-curve as an outlier. It turned out that it is difficult to detect outliers because point-based results of these analyses did not differ from those not considered as outliers. What did differ was the whole pattern of response, shown as an S curve, obtained within a specific period just before and just after the occurrence of the stress event. Further reasoning led us to the observation that these S curves might not necessarily be an outlier but may to appear as a regular outcome of the experiment (see reasoning below).

In the second step, we assumed that the challenge might be solved by applying a larger sample size. In empirical research, however, obtaining physiological responses in experimental settings from larger samples of experienced marine pilots is impractical. Instead, we increased the number of participants by simulating the data rather than collecting new empirical data; the simulations were based on real data obtained in a previous experiment. Continuous autoregressive signal generation was used for the simulation. The simulated model was estimated from the real data using Python library TimeSynth. To test the hypothesis that data set enlargement might solve the challenge of identifying and treating the dominant curves (the determinators), we ran the simulation on a sample which could be considered a large sample for physiological research, 109 participants.

Our simulations showed that more than a simple increase in sample size is needed to solve the issues regarding what we considered an outlier. Both simulations indicated that a small data set might determine the response pattern relatively independently of the sample size. Data sets that matched the S-curve with relatively high amplitudes were likely to determine an averaged response curve.

In the third step, we introduced bootstrap analysis as a technique to identify a possible dominant curve (determinator) hidden in the data. Bootstrapping is a resampling method (sampling with replacement) where a given sample (set of signals) is subsampled, and characteristics are estimated from these subsamples. Typically, it is used to measure the accuracy of estimators (bias, variance, confidence intervals, etc.) (Efron, 1979; Davison & Hinkley, 1997) and selected characteristics of underlying distributions. Bootstrapping was applied to identify the presence of shape-dominant curves by plotting histograms of signals’ total variation. The results showed that the presence of a dominant curve can be identified by the bimodality or the polymodality of the histograms.

The bootstrapping estimation method of total variations (TV) is a promising method that can help us identify subgroups of characteristic determining shapes in our set of signals. In short, unimodal histograms indicate a unimodal (homogenous) group of curves, and a multimodal histogram indicates several groups and heterogeneous types of data, which may alert researchers of the need for further inspection. The conclusions derived from the means are likely reliable when a single group is shown on a histogram.

Note that an alternative approach to identification of misleading results is using the appropriate clustering method. Here, the characteristic S curves would present one cluster, and the rest of the curves would represent the rest of the clusters. However, our attempts to identify characteristic curves did not yield promising results. This might be due to the distance among non-characteristic curves which tends to be large and comparable in size to the distance of S curves to the rest of curves. A proper normalization of curve ranges did not improve results significantly. Therefore, we conclude that the clustering approach may not be appropriate for the misinterpretation problem identification.

The likelihood of the misinterpretation being based on mean curves as presented in this paper is higher than one would expect, and this makes our observation relevant for a wider audience. Why is it higher? The misinterpretation of a mean curve arises only when a small number m of S curves appears in the sample compared to the sample size n. For larger m compared to n, the impact of S curves to the mean S curve is a correct impact and thus not a misinterpretation. When the probability of S-shape appearance is denoted by p , the skewness of the underlying geometrical distribution (see section 3.2.3) is (2-p)/√(1-p) and it approaches to two for small p and approaches to infinity for p getting close to one. In our case, the skewness is always above two and the distribution is leaning toward low probabilities. Therefore, the probability of getting a low number of S curves is relatively large, meaning that such curves may not be outliers, but may appear as a rule in the real data and that the likelihood that the problem of misinterpretation arising in real experiments is high.

In our experiment there were 8 experienced pilots and 8 students. The estimation of probability of appearance of S-curve for inexperienced pilots was higher than for experienced ones as expected.

Note, however, the generation modeling by geometrical distribution is based on the assumption that that the probability of stress response of the participant is independent of previous stress events. This assumption might be addressed in greater detail in future research.

## 6 Conclusion

Advanced statistical methods and the ubiquitous use of statistics in not just the physical sciences, but the humanities as well, have perhaps led to a degree of excessive trust in what they appear to communicate. Previous work regarding physiological signals in the study of the stress undergone by maritime pilots indicated that the statistical presentation of stress factors obviously did not align with what could clearly be seen by researchers on hand during the testing. Maritime pilots have one of the best-paid but riskiest jobs in the transportation industry, engaged in an extremely demanding job often carried out in very difficult conditions with the stress of an awareness that a mistake can lead to serious accidents, loss of life, loss of goods, environmental damage, etc. These circumstances illustrated the need to understand statistical results better and to be aware of the hidden anomalies that statistically precise results might deliver.

Our analysis showed that a single participant or a small data set can determine results, leading the researcher to a biased conclusion. This mechanism is not due to a small sample size. The results of our simulations indicated that a plain increase in data sets (or the number of participants) would not solve the challenge of determining to what degree statistical presentations might or might not be trustworthy. Is an outlier determining the results, which might lead to inaccurate conclusions about pilots’ heart rate or electrodermal activity response preceding stress events during the port approach?

Classical statistical methods to identify outliers do not work well because what we may consider outliers are determined by a complex pattern series response rather than point-related data sets that are likely to escape statistical detection. In addition, a detailed visual inspection may also fail to identify anomalies because time series visualizations depend heavily on scales and temporal periods included in the inspection; in our case, the visualizations depended on how we defined the beginning of the stress event (to anchor the curves), as well as to the periods just before and just after a stress event included in the analyses. This kind of visualization technique requires both experience and intuition to detect hidden patterns behind small-scale repeated measurements and large time-related data sets such as heart rate or electrodermal activity during the port approach.

This article was written to alert researchers to the problem of over-relying on statistical “results.” As a byproduct, attention is brought to the specific case of studying physiological signals in simulated environments, and in particular, the study of maritime pilots. This is particularly important because physiological measurements are becoming increasingly accurate, so researchers are making advances using various experiments with real-time data analysis on simulators and in the real-world environment.

## Data Availability

The raw data supporting the conclusions of this article will be made available by the authors, without undue reservation.

## Footnotes

^1^ https://pypi.org/project/timesynth/
